# Evaluation and Correction for Optical Scattering Variations in Laser Speckle Rheology of Biological Fluids

**DOI:** 10.1371/journal.pone.0065014

**Published:** 2013-05-21

**Authors:** Zeinab Hajjarian, Seemantini K. Nadkarni

**Affiliations:** Wellman Center for Photomedicine, Massachusetts General Hospital, Harvard Medical School, Boston, Massachusetts, United States of America; Tufts University, United States of America

## Abstract

Biological fluids fulfill key functionalities such as hydrating, protecting, and nourishing cells and tissues in various organ systems. They are capable of these versatile tasks owing to their distinct structural and viscoelastic properties. Characterizing the viscoelastic properties of bio-fluids is of pivotal importance for monitoring the development of certain pathologies as well as engineering synthetic replacements. Laser Speckle Rheology (LSR) is a novel optical technology that enables mechanical evaluation of tissue. In LSR, a coherent laser beam illuminates the tissue and temporal speckle intensity fluctuations are analyzed to evaluate mechanical properties. The rate of temporal speckle fluctuations is, however, influenced by both optical and mechanical properties of tissue. Therefore, in this paper, we develop and validate an approach to estimate and compensate for the contributions of light scattering to speckle dynamics and demonstrate the capability of LSR for the accurate extraction of viscoelastic moduli in phantom samples and biological fluids of varying optical and mechanical properties.

## Introduction

Biological fluids like synovial fluid, vitreous humor, cerebrospinal fluid, blood, lymph, and mucus are biopolymer solutions of water, protein macromolecules, and cells [1], [2]. They serve as shock-absorbers, allergen and bacteria trappers, nutrient and oxygen distributers, and lubricants in different organ systems [2]–[6]. To fulfill these roles, bio-fluids maintain distinct viscoelastic behavior, exhibiting both solid and fluid-like features under different loading conditions and size scales [3]–[7]. Disease progression in multiple organ systems is frequently accompanied by altered viscoelastic properties of bio-fluids. For instance, in rheumatoid arthritis and osteoarthritis, the reduction of glycosaminoglycan hyaluronate and lubricin contents alters the viscoelastic properties of synovial fluid, compromising its shock-absorbing capability, in turn damaging cartilage under loading condition [8]. The abundance of evidence on the reduced viscosity of synovial fluid in the course of osteoarthritis has led to development of Viscosupplementation, a treatment approach in which diseased synovial fluid is replaced with an elastoviscous hyaluronan solution [9]. In the case of another bio-fluid, vitreous humor, altered viscoelastic properties are believed to be associated with age and numerous ocular pathologies such as retina tear and detachment [6], [10]. The significant evidence on the role of bio-fluid viscoelasticity in disease initiation and progression, therefore, calls for the development of novel technologies for mechanical evaluation of bio-fluids in their native state to advance our understanding of bio-fluid pathologies, improve clinical disease diagnosis and facilitate the development of treatment strategies.

The viscoelastic modulus, G*(ω) = G′(ω)+i G″(ω), defines the mechanical behavior of materials. It is traditionally measured using a mechanical rheometer by evaluating the ratio of a shear oscillatory stress at frequency *ω*, exerted upon the sample to the induced strain. The elastic modulus, *G′(ω)*, is the real part of G*(ω) and defines the energy stored in the sample. The viscous modulus, *G″(ω)*, is the imaginary part and represents viscous dissipation of the material 2,11. The moduli G*, *G′* and *G″* are often expressed in the units of Pascal's (Pa). For instance, the typical values of |G*(ω)| for soft tissues (at *ω* = 1 Hz) are 60 m Pa for blood, 1 Pa for vitreous humor, 1.6 K Pa for fat, and 4.5 K Pa for muscle [6], [12].

Laser Speckle Rheology (LSR) is a new optical approach that measures the viscoelastic properties of samples in a non-contact manner and holds the potential for evaluating the viscoelastic properties of tissues *in situ*, in their native states [12]–[16]. In LSR, the sample is illuminated with coherent laser light and the time-varying speckle intensity fluctuations are recorded using a high speed camera. Temporal speckle intensity fluctuations are exquisitely sensitive to the mean square displacement (MSD) of light scattering centers undergoing Brownian motion, and the extent of this thermal motion reflects the viscoelastic properties of the surrounding medium [17]–[26]. To quantify the rate of speckle fluctuations, the correlation coefficient between successive speckle frames is measured over time to obtain the speckle intensity temporal autocorrelation curve, *g_2_(t)* [27]–[30]. The MSD of light scattering centers (also denoted as *<Δr^2^(t)>* in equations and figures throughout this paper) can be estimated from *g_2_(t)* [31]–[35], and the Generalized Stokes'-Einstein Relation (GSER) is used to deduce the viscoelastic modulus, G*(ω) from the measured MSD [18]–[20], [22], [23], [25], [26], [36]–[41].

A major challenge is that temporal speckle intensity fluctuations, given by *g_2_(t)* curve, not only depend on the viscoelastic properties, but are also intimately influenced by optical properties of the medium, particularly by optical scattering [33], [42], [43]. Traditionally, in the limits of single scattering or strong multiple scattering (diffusive regime) media, dynamic light scattering (DLS) and diffusing wave spectroscopy (DWS) formalisms have been used to extract MSD from the measured *g_2_(t)* curve [17], [19], [21]–[23], [28], [44], [45].

Estimating G*(ω) of bio-fluids that do not meet the limits of single scattering or diffusive regime, however, is not straightforward. Bio-fluids span a range of optical properties and may be weakly, moderately, or highly scattering. Moreover, pathologies often simultaneously alter both optical and mechanical properties of bio-fluids, further confounding the accurate estimation of viscoelastic properties using LSR [46]. In this paper, we show that for a majority of turbid media, that do not meet the limits of single scattering or diffusion approximations, DLS and DWS formalisms lead to erroneous measurements of MSD, and in turn result in inaccurate moduli estimates. We demonstrate that in order to accurately measure bio-fluid viscoelasticity, the influence of light scattering must be decoupled from that of mechanical properties in interpreting the speckle dynamics and *g_2_(t)*. We therefore introduce a novel approach to improve the accuracy of LSR for the mechanical characterization of bio-fluids of varying optical properties by correcting for the influence of arbitrary optical scattering. In this approach, we measure sample optical properties from time-averaged speckle patterns, and implement a polarization sensitive correlation transfer Monte-Carlo ray tracing (PSCT-MCRT) algorithm to correct for the contribution of optical scattering in MSD evaluation. The close correspondence between LSR measurements and conventional rheology of phantom and bio-fluid samples, presented below, establishes the capability of the new approach in accurately evaluating the viscoelastic modulus, G*(ω), for biological fluids of arbitrary optical properties.

## Materials and Methods

### Sample preparation

The studies below were performed using glycerol and bio-fluid samples. Various Glycerol-Water mixtures (G-W) were prepared at different proportions (60%G-40%W, 70%G-30%W, 80%G-20%W, 90%G-10%W, and 100%G) over a range of viscosities (0.01–1.4 Pa. s). Frozen bovine synovial fluid and vitreous humor (Animal Technologies, Tyler, TX) were warmed up to 37°C in a water bath prior to LSR testing. The glycerol and bio-fluid samples were chosen for their optical clarity, which allowed us to validate our approach via tuning their scattering properties by adding various concentrations of TiO_2_ particles. In all cases, TiO_2_ particles (dia. ∼400 nm, Anatase, Acros organics, Belgium) were added to glycerol mixtures in multiple concentrations (0.04%–2% volume fractions, corresponding to reduced scattering coefficients, *μ′_s_*, : 1.3–84.8 mm^−1^, N = 18) and thoroughly mixed in a vortex to ensure even dissemination of scattering particles. For both phantoms and bio-fluids, 1.5 ml of the samples were placed in a clear cuvette (Fischer brand, light path 10 mm, 1.5 ml) for LSR measurements, and 2 ml were used for mechanical testing. The LSR approach described here will be used in the future to evaluate the viscoelastic properties of bio-fluids in their native state, without adding extrinsic scattering particles. However, in this study, extrinsic TiO_2_ particles were utilized purely for the purpose of validating our approach over a large range of optical scattering concentrations relevant to tissue.

### LSR optical setup

**Figure 1:**
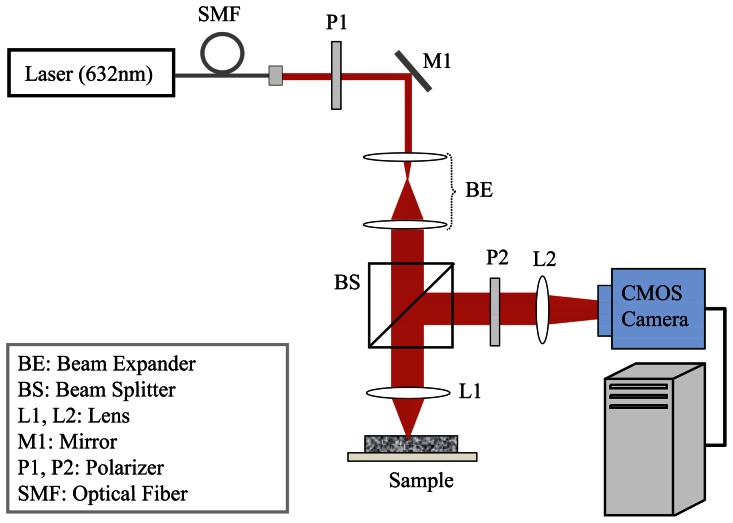
Schematic of the LSR optical setup [12]–[14], [16]. Light from a randomly polarized He-Ne laser (632 nm, 30 mW) is coupled into a single mode fiber (SMF600). The beam is polarized, collimated, and focused (focal length 25 cm, 50 µm spot size) at the sample surface. A beam-splitter is used to ensure speckle patterns are acquired at 180° back-scattering geometry. The cross-polarized component of back-scattered light is focused at the CMOS sensor of a high-speed camera (PL-761, Pixelink, Ontario, Canada), equipped with a focusing lens system (MLH-10×, Computar, Commack, NY). The acquired speckle frame series are transferred to a high-speed computer for further processing.

Laser speckle frame series of samples were acquired using the optical setup shown in Fig. 1 [12]–[14], [16]. Light from He-Ne Laser (633 nm) was coupled into a single mode fiber (SM600), and focused to a 50 µm spot on the sample. Cross-polarized laser-speckle patterns were acquired at 180° backscattering geometry via a beam-splitter using a high frame rate CMOS camera (PL-761, Pixelink, Ontario, Canada).

### Measurement of speckle intensity temporal autocorrelation curves from time-varying speckle patterns

**Figure 2:**
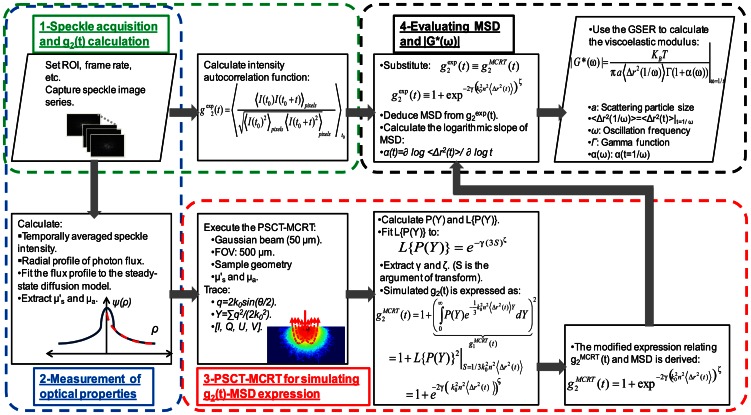
Detailed flow chart of the compensation algorithm. *Block 1: Speckle acquisition and g_2_(t) calculation:* Speckle frame series are acquired with sufficient frame rate, ROI, and pixel to speckle size ratio. Speckle intensity temporal autocorrelation curves, *g_2_(t)*, are evaluated for phantom and tissue samples using sufficient temporal and spatial averaging. *Block 2: Measurement of optical properties*: The radial remittance profile is evaluated from temporally averaged speckle intensities and is converted to the photon flux, *ψ(ρ)*. Optical properties of the sample (*μ_a_* and *μ′_s_* ) are derived experimentally by fitting the photon flux profile to the model obtained from steady-state diffusion theory. *Block 3: PSCT-MCRT for simulating g_2_(t)-MSD expression:* Experimentally evaluated optical properties, LSR configuration, and sample geometry are used in the PSCT-MCRT simulation to derive an expression for *g_2_(t)* as a function of MSD. *Block 4: Evaluating MSD and* |G*(ω)|: Following the measurement of MSD using the modified expression, logarithmic slope of MSD, *α(t) = ∂ log <Δr^2^(t)>/∂ log t*, is calculated and replaced in the simplified GSER to evaluate the viscoelastic modulus [18]–[20], [22], [23], [25], [26], [36]–[41]. Here *K_B_* is the Boltzman constant (1.38×10^−23^), *T* is temperature (degrees kelvin), *a* is the scattering particle size, *<Δr^2^(1/ω)>* corresponds to *<Δr^2^(t)>*, evaluated at t = 1/ω, ω = 1/t is the frequency, and *Γ* represents the gamma function.

For glycerol samples, time-varying speckle images were captured for 2 second at 490 frames per second (fps) (Fig. 2). Speckle size was adjusted to at least twice the pixel size (12 µm) to maintain sufficient spatial sampling and contrast. For bio-fluids, a higher frame rate (840 fps) was used due to relatively faster speckle dynamics. The speckle intensity temporal autocorrelation curve, *g_2_^exp^(t)*, was obtained by measuring the correlation coefficient of pixel intensities in the first speckle image (time point *t_0_*) with subsequent images (time points *t_0_+t*, 0≤*t*≤2) in the image series, over a 2 s duration (Fig. 2, Block 1). Spatial averaging was performed over 40×40 pixels, and several *g_2_(t)* curves evolving in time were averaged to enhance the accuracy of temporal statistics as follows [12]–[16], [19], [22], [23], [35], [47]:
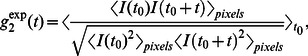
(1)where *I(t_0_)* and *I(t+t_0_)* referred to the speckle images at times *t_0_* and *t+t_0_*, *< >_pixels_* and *< >_t0_* indicated spatial and temporal averaging over all the pixels in the images and for entire imaging duration (2 s), respectively.

### MSD evaluation using DLS and DWS Formalisms

For single or strong multiple scattering media, DLS and DWS theories, respectively, have expressed the measured *g_2_(t)* (eqn. (1)) as a function of MSD as below [17], [21], [32], [44], [48], [49]:
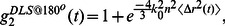
(2)

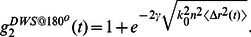
(3)Here *k_0_* is the wave number, *n* is the refractive index of the sample, and *γ* is an experimental parameter that is generally assumed to be equal to 5/3 (∼1.7) [17]–[24], [27], [28], [31], [44], [45]. Since use of the DLS formalism is not valid for the moderate to strongly scattering samples used in this paper, the accuracy of our new LSR approach for measuring sample viscoelasticity is compared with that obtained using the DWS formalism (eqn. (3)) as described below.

### Algorithm to derive corrected MSD and measure G*(ω) using LSR

To correct for the influence of arbitrary optical scattering in samples that do not meet the criteria of single scattering or light diffusion regimes, we have developed an algorithm that utilizes experimentally measured optical properties in a PSCT-MCRT model to establish a modified expression for *g_2_(t)* which corrects for influence of optical scattering in MSD calculations. The steps involved in measuring and correcting for optical scattering, the computational methods employed in the algorithm, and estimation of MSD and G*(ω) in LSR are shown in the flowchart (Fig. 2) and detailed below.

#### i) Experimental evaluation of optical properties from time-averaged speckle images

Optical properties were derived experimentally by measuring the radial remittance or photon flux profile of samples from time-averaged speckle images using previously published methods (Fig. 2, Box 2) [14], [50]–[55]. Briefly, the speckle image series were temporally averaged over an ROI of 296×296 pixels at the CMOS sensor (Field of View (FOV) of 2 mm). The average pixel intensity values were converted to photon flux based on camera responsivity (28.1 DN/(n J/cm2)), gain (12.04 dB), exposure time (1 ms), and the (solid) angle of view. The radial photon flux profile was then fitted to the model predicted by the steady-state diffusion theory to evaluate the absorption and reduced scattering coefficients (*μ_a_*, *μ′_s_*) [14], [51]. Since the asymmetry parameter, *g*, is not trivially related to photon flux, the value of *g* and the corresponding scattering phase function were calculated from Mie theory predictions, which resulted in *g* = 0.6 for TiO_2_ particles suspended in glycerol solutions (see Discussion) [56]. For predominantly scattering samples in this study, the optical absorption coefficients, *μ_a_*, were negligible. The accuracy of this approach in estimating *μ′_s_* for glycerol and bio-fluid samples with varying TiO_2_ concentrations was confirmed via comparison with Mie theory estimates [56].

#### ii) PSCT-MCRT simulation to establish the modified g_2_(t) and MSD relationship

The PSCT-MCRT algorithm below was employed to simulate *g_2_^MCRT^(t)* curves (Fig. 2, Box 3) and derive a modified relationship between MSD and *g_2_(t)*, for samples with arbitrary optical properties. The PSCT-MCRT model incorporated all experimental LSR parameters, for a focused Gaussian beam (50 µm) illuminating the sample placed in a cuvette (10 mm light path, 1.5 ml) with *μ_a_* and *μ′_s_* measured as above. A total of 10^5^ photons were tracked from the source to the receiver (FOV of 2 mm). The temporal speckle fluctuations were modified by the polarization state of detected light. Therefore, PSCT-MCRT algorithm incorporated attributes of the polarization state by tracking the Stokes' vector, *[I Q U V]*, with respect to the corresponding reference frame. Euler equations were used to modify the Stokes' vector upon scattering and transport within the medium [57], [58]. At the receiver site, a final rotation was applied to redefine the Stokes' vector in the receiver coordinates system and since LSR setup captured the rapidly evolving speckle pattern of the cross-polarized channel (Fig. 1), only the cross-polarized component of intensity was retained. To account for momentum transfer (Doppler shift) at each scattering event, the scattering wave vector, *q = 2k_0_sin(θ/2)*, was tracked, as well [17], [35]. The total momentum transfer, defined as *Y = ∑q^2^/(2k_0_^2^)*, with the summation over all scattering events involved in that path, represented the reduction of speckle intensity temporal autocorrelation due to all scattering events involved in each path [35], [59]. Consequently, *g_2_^MCRT^(t)* was obtained by integrating the field temporal autocorrelation curves of all received rays, weighted by the corresponding momentum transfer distribution, *P(Y)*, as [43], [60]:
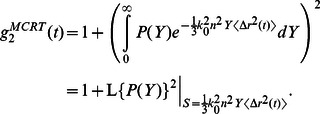
(4)From eqn. (4), it is noted that the term in brackets is simply the Laplace transform of *P(Y)*, *L{P(Y)}* evaluated at *1/3k_0_^2^n^2^<Δr^2^(t)>* [43], [61]. *L{P(Y)}* is equivalent to speckle field autocorrelation, *g_1_^MCRT^(t)*, in turn related to speckle intensity autocorrelation curve, *g_2_^MCRT^(t)*, through the Siegert relation as: *g_2_(t) = 1+|g_1_(t)|^2^* [27], [28], [35], [44], [62]. PSCT-MCRT only provided the statistical histogram of photons' *P(Y)* and generated a numerical solution for *L{P(Y)}* ( = *g_1_^MCRT^*(*t*)), and consequently *g_2_^MCRT^(t)*. To simplify eqn. (4), a parametric function was fitted to *L{P(Y)}* as below:
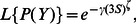
(5)where *S* was the argument of the transform (complex frequency). The parameters *γ* and *ζ* were derived from PSCT-MCRT simulation by numerical calculation of the total momentum transfer distribution *P(Y)*, based on the experimentally evaluated values for *μ_a_* and *μ′_s_* and Mie theory calculations of *g* for each individual sample. Consequently, the following expression was derived for *g_2_^MCRT^(t)* as a function of MSD (see Discussion):
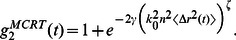
(6)


The parametric functions of eqns. (5) and (6) were cross-checked for the limits of single scattering and diffusion approximations (eqns. (2) and (3)). For instance, for TiO_2_ concentration of 0.04%, and *μ′_s_* = 1.3 mm^−1^, the results of PSCT-MCRT gave rise to empirical parameters of *γ* = 2/3 and *ζ* = 1, in which case eqn. (6) above converged to the DLS expression of eqn. (2). On the other hand, for TiO_2_ concentration of 2%, corresponding to *μ′_s_* value of 84.8 mm^−1^, MCRT results gave rise to *γ* = 5/3 and *ζ* = 0.5, and the eqn. (6) converged to the DWS formalism (eqn. (3)). Since *γ* and *ζ* are directly related to the sample optical properties, the need for repetitive execution of MCRT simulations can be eliminated by calculating the *γ* and *ζ* parameters for a wide range of *μ_a_* and *μ′_s_* values relevant to tissue, beforehand, and preserve the trends in a look up table (LUT). Thus, in the future by measuring the *μ_a_* and *μ′_s_* of the sample, the corresponding *γ* and *ζ* parameters can be simply obtained from the LUT without the need for executing PSCT-MCRT simulations.

#### iii) Estimating the compensated MSD and evaluating G*(ω)

Eqn. (6) above established the general expression relating MSD with *g_2_(t)* over a range of optical scattering concentrations that span the limits of single scattering and light diffusion regimes. By substituting *g_2_^MCRT^(t)* (from eqn. 6) with *g_2_^exp^(t)* (from eqn. 1), and using parameters *γ* and *ζ* obtained from PSCT-MCRT simulations, the corrected MSD values of samples were deduced (Fig. 2, Box 4). In a purely viscous medium, the Stokes'-Einstein equation relates the diffusion coefficient of particles of known radius, with the viscosity of the material [63]. Mason, Weitz, and others have developed a formalism that generalizes the Stokes'-Einstein equation and relates the particular MSD with the modulus, G*(ω), of viscoelastic materials with more complex frequency-dependent mechanical behavior (eq. 7) [18]–[20]. By applying this generalized Stokes'-Einstein relation (GSER), in the final step of our algorithm, MSD was used to extract G*(ω) (Fig. 2, Box 4) [18]–[20], [22], [23], [25], [26], [36]–[41]. To this end logarithmic slope of MSD was calculated and replaced in the simplified GSER to evaluate the viscoelastic modulus [18]–[20], [22], [23], [25], [26], [36]–[41]:
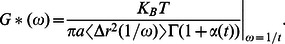
(7)Here *K_B_* is the Boltzman constant (1.38×10^−23^), *T* is temperature (degrees Kelvin), *a* is the scattering particle radius, *<Δr^2^(1/ω)>* corresponds to *<Δr^2^(t)>*, evaluated at *t = 1/ω*, *ω = 1/t* is the frequency, *Γ* represents the gamma function, and *α(t) = ∂ log <Δr^2^(t)>/∂ log t|_t = 1/ω_* is the logarithmic slope of MSD.

### Comparison of LSR with reference-standard mechanical testing

To validate our approach above, we compared LSR measurements of the magnitude of viscoelastic moduli, |G*(ω)|, with those obtained using a reference standard mechanical rheometer (AR-G2, TA Instruments, MA). To conduct mechanical rheometry, the rotating rod and top plate (40 mm dia. stainless steel) exerted a shear oscillatory torque (stress) upon the sample over the frequency range of 0.1–100 Hz and measured the induced strain. For aqueous glycerol mixtures, tests were carried out at 25°C, and 2% strain was applied on the samples. Synovial fluid and vitreous humor samples were evaluated at 37°C with a 1% strain. The accuracy of our new modified approach in measuring the viscoelastic moduli of glycerol and bio-fluid samples was further compared with that obtained using conventional DWS (eqn. (3)).

## Results

### Influence of optical scattering on LSR measurements

**Figure 3:**
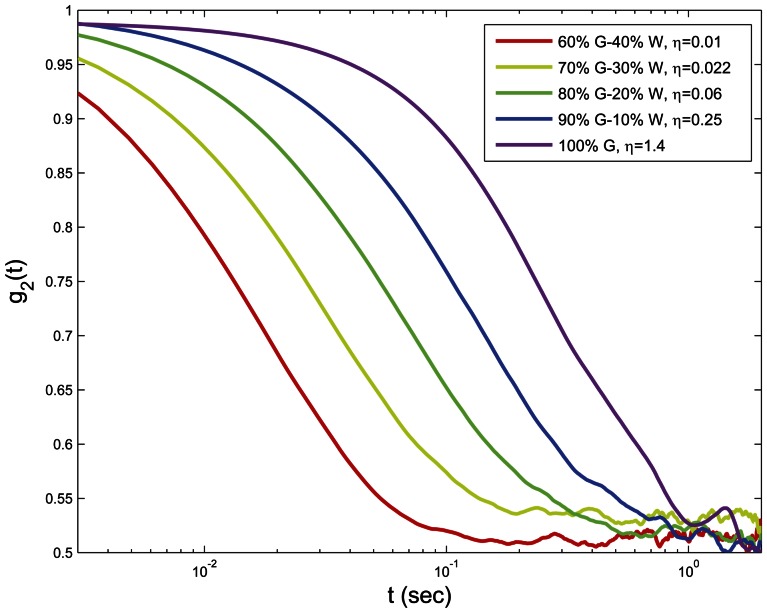
LSR of aqueous glycerol mixtures of different viscosities. Speckle intensity temporal autocorrelation curves, *g_2_^exp^(t)*, for aqueous glycerol mixtures (100% G, 90%G-10%W, 80%G-20%W, 70%G-30%W, and 60%G-40%W) with 0.1% volume fraction TiO_2_ scattering particles. It is observed that for higher viscosity liquids speckle intensity temporal autocorrelation decays slower.

**Figure 4:**
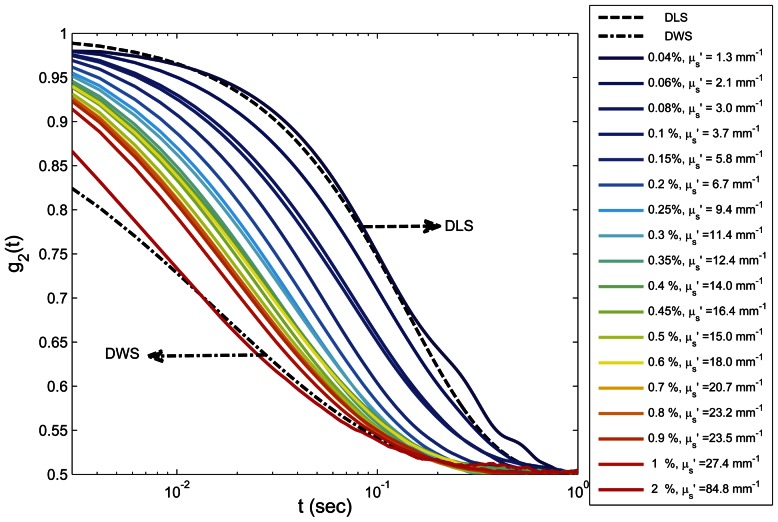
LSR of 90% glycerol-10% water mixtures with varying scattering concentrations. Speckle intensity temporal autocorrelation curves, *g_2_^exp^(t)*, for aqueous glycerol mixtures of 90%G-10%W and various concentrations of TiO_2_ scattering particles (0.04%–2%, corresponding to *μ′_s_ : 1.3–84.8 mm^−1^*, N = 18), along with theoretical DLS and DWS curves (dotted lines). By changing the scattering concentration *g_2_^exp^(t)* curves sweep the transition area between the two theoretical limits. This data demonstrates the dependence of *g_2_^exp^(t)* on optical scattering in samples with identical mechanical properties.

**Figure 5:**
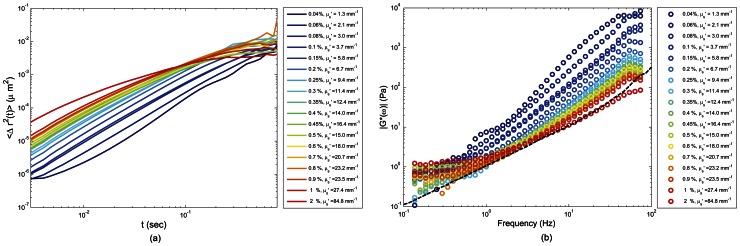
MSD of scattering particles, derived using DWS expression, and the corresponding magnitude viscoelastic modulus |G*(ω)| curves for 90% glycerol-10% water mixtures. In panel (a), MSD is extracted from *g_2_^exp^(t)* assuming the validity of Diffusion approximation. Considerable variability is observed between MSD curves associated with different scattering concentrations. In panel (b) Generalized Stokes'-Einstein Relation is used to calculate |G*(ω)| from MSD obtained from Diffusion approximation. The curves fail to match the results of conventional rheometry and are biased by the corresponding scattering concentrations. Moreover, significant variation is observed between the evaluated modulus of sample with different scattering concentrations.

Fig. 3 shows the speckle intensity temporal autocorrelation curves, *g_2_^exp^(t)* for glycerol-water (G-W) mixtures of varying viscosities with 0.1% of TiO_2_ scattering particles. As expected, for liquids of higher viscosity, *g_2_^exp^(t)* curves decayed slower due to reduced Brownian motion of TiO_2_ particles compared to the lower viscosity samples. Here, given the identical optical properties of samples (0.1% TiO_2_), a direct comparison of *g_2_^exp^(t)* curves enabled an accurate assessment of relative differences in sample viscosities. However, variation in scattering concentrations also modified speckle dynamics even in samples with identical viscosity, as demonstrated in Fig. 4, which displays measured *g_2_^exp^(t)* curves for 90% G - 10% W (volume fraction) with viscosity ∼0.25 Pa. S. While these samples had identical mechanical properties, optical scattering properties were tuned by mixing with different TiO_2_ particle concentrations (0.04%–2%). To ensure that the addition of TiO_2_ particles did not affect the sample mechanical properties, the viscoelastic moduli of glycerol suspensions with different TiO_2_ particle concentrations (up to 2%) were measured. No detectable differences in G* were measured even at the highest TiO_2_ concentration. Fig. 4 also displays the *g_2_(t)* curves obtained from eqns. (2) and (3) based on DLS and DWS formalisms (dashed and dot-dashed lines). From Fig. 4 it was evident that for most scattering concentrations, *g_2_^exp^(t)* curves fell somewhere between the dotted curves. In other words, by changing the TiO_2_ concentration from 0.04% to 2%, *g_2_^exp^(t)* curves swept the gap between theoretical limits of single and multiple scattering, demonstrating a dramatic change in temporal speckle intensity fluctuations for samples of identical viscosities. Further, the results that follow established that the direct usage of DWS approximation for extracting the MSD of scattering particles caused erroneous estimation of |G*(ω)|. The results of exploiting DLS formalism were not presented here, since most of our samples were visibly not dilute enough to be considered single scattering. Fig. 5(a) shows the emerging errors by displaying the measured MSD, from *g_2_^exp^(t)* curves of Fig. 4, using the DWS approximation for backscattering geometry (eqn. (3)) [31], [35]. As expected, a large variation was observed between the curves and scattering dependence in the *g_2_^exp^(t)* plots were directly conveyed to MSD curves. Fig. 5(b) displays the resultant |G*(ω)| curves, calculated by substituting the raw MSD curves of Fig. 5(a) in the GSER (eqn. (7)) and the |G*(ω)| measured using the rheometer (dashed line) [18]–[20], [22], [23], [25], [26], [36]–[41]. Results of Fig. 5(b) were clearly biased by variations in scattering concentrations and failed to correspond with conventional rheology results (dashed line), for most curves. In particular, |G*(ω)| was over estimated using DWS formalisms, especially at lower concentrations, due to slower decay of the g_2_(t) curve, influenced by lower optical scattering independent of viscoelastic properties. It was only at TiO_2_ concentration of 2%, that strong multiple scattering dominated, the diffusion approximation was valid, and |G*(ω)| approached the results of mechanical rheometry.

### Results of LSR using the new optical scattering correction algorithm

#### i) LSR results for glycerol suspensions

**Figure 6:**
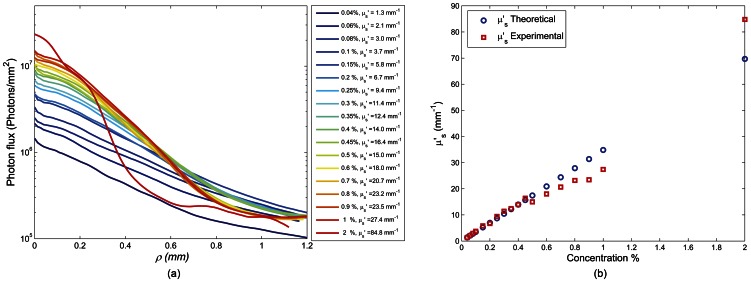
Radial photon flux profile of 90% glycerol-10% water mixtures with varying scattering concentrations and the corresponding theoretical and experimental estimation of the reduced scattering coefficient, *μ′_s_*. Panel (a) shows the photon flux profile of the glycerol suspensions. It is observed that for samples of higher scattering particles' concentration, the net backscattered signal level increases. At the same time, the curves decay faster as a function of radial distance. Transport albedo (*μ′_s_*/(*μ′_s_+μ_a_*)) and effective attenuation coefficient (√*μ_a_* (*μ′_s_+μ_a_*)) are derived by fitting the photon flux to theoretical models of the steady-state diffusion theory to further extract *μ′_s_* and *μ_a_* [51]. In panel (b) Mie theory estimates of *μ′_s_* are shown, which are derived based on TiO_2_ particle size, source wavelength, and the ratio of refractive indices of TiO_2_ particles and glycerol solutions(refractive index mismatch). A close correspondence is observed between experimental and theoretical measurements of the *μ′_s_* (*R = 0.96, P<0.0001*), especially at lower scattering concentrations. For higher scattering concentrations, potential sedimentation of scattering particles, and particle interactions lead to distortion of photon flux curves and saturation of evaluated parameters.

Fig. 6 demonstrates the validity of our methods for evaluating the optical properties of phantom glycerol samples from time-averaged speckle images. Fig. 6(a) shows the radial profile of photon flux measured as a function of distance from the illumination center for the glycerol suspensions of 90%G-10%W with TiO_2_ scattering concentrations ranging from 0.04%–2%. In Fig. 6(a) the number of remitted photons per unit area (photon flux) intensified at higher concentration and the inset of curves increased while slope of the photon flux profile became steeper. *μ′_s_* and *μ_a_* were derived by fitting the photon flux profile (Fig. 6(a)) to theoretical models of the steady-state diffusion theory [14], [51]. Fig. 6(b) shows the experimentally evaluated *μ′_s_* values plotted against corresponding Mie theory predictions [56]. Good agreement was observed (*R = 0.96, p<0.0001*), demonstrating the validity of the experimental approach in assessing sample optical properties. The results were more accurate for low to moderately scattering samples but started to diverge at higher concentrations as discussed below.

**Figure 7:**
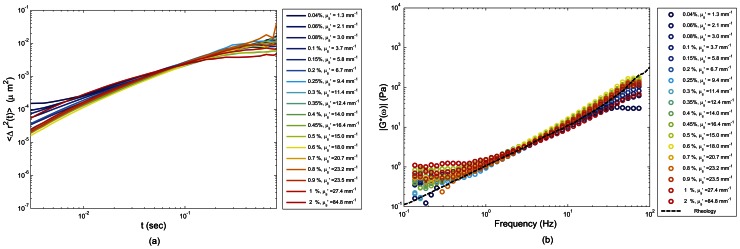
Compensated MSD of scattering particles and the corresponding magnitude viscoelastic modulus, |G*(ω)|, of 90% glycerol-10% water-TiO_2_ suspensions. Panel (a) depicts the corrected MSD curves, deduced from *g_2_^exp^(t)* curves of Fig. 4 using eqn. (6). The modified expression of eqn. (6) resulted from PSCT-MCRT simulation of photon propagation and correlation transfer in LSR experimental setup considering the exact sample geometry and optical properties. Compared to Fig. 5(a), variability of MSD curves is significantly reduced, especially at intermediate times. Residual small deviations, still observed at very early or long times, are most likely due to electronic noise and speckle blurring, respectively. In panel (b) Generalized Stokes'-Einstein Relation is used to calculate |G*(ω)| from corrected MSD. It is observed that the variability between measured |G*(ω)| for different concentrations is considerably reduced, compared to Fig. 5(b). Moreover, a high correspondence is observed between LSR results for |G*(ω)| and mechanical rheometry.

Fig. 7(a), plots the MSD of particle dynamics in glycerol suspensions of 90%G-10%W, measured by employing the PSCT-MCRT based optical scattering correction algorithm. As noted, the variability between MSD curves over the range of scattering concentrations (0.04%–2%), was significantly reduced compared to Fig. 5(a) (which employed the DWS formalism). The impact of corrections was more pronounced in the intermediate times and residual small deviations were still observed at very early or long times, corresponding to initial decay and final plateau of *g_2_^exp^(t)*. These mismatches were most likely due to certain experimental factors, as discussed later. Fig. 7(b) showed the LSR evaluation of |G*(ω)| for the 90%G-10%W samples measured by employing optical scattering compensation compared with the corresponding rheometer measurements (dashed line). Compared to Fig. 5(b), the optical scattering dependence of |G*(ω)| curves was significantly reduced by employing the compensation algorithm. Moreover, the scattering compensated moduli corresponding to all scattering concentrations closely corresponded with the measurements of mechanical rheometer. Our results showed that while differences in optical properties dramatically modulated the *g_2_(t)* curves, a significant improvement was achieved in the LSR evaluation of viscoelastic moduli, by compensating for optical scattering variations versus the direct application of DWS formalism in the estimation of MSD, and calculation of the |G*(ω)|.

#### ii) LSR results of biological fluids

**Figure 8:**
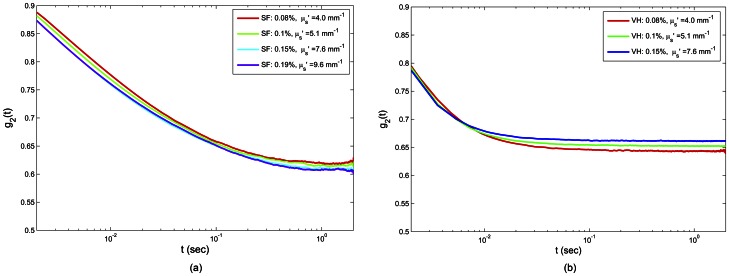
Speckle intensity temporal autocorrelation curves of synovial fluid and vitreous humor. Panel (a) depicts the measured *g_2_(t)* curves of synovial fluid samples mixed in with TiO_2_ particles of different concentrations (0.08%, 0.1%, 0.15%, 0.19%, corresponding to *μ′_s_ :4.0, 5.1, 7.6, and 10.1 mm^−1^*, respectively), and panel (b) displays the curves corresponding to vitreous humor samples mixed in with TiO_2_ particles (0.08%, 0.1%, 0.15, corresponding to *μ′_s_ :4.0, 5.1, and 7.6 mm^−1^*, respectively). It is observed that early decay accelerates by increasing the scattering coefficient. At longer times, there is an artificial increase of the curve plateau level due to blurring of rapidly fluctuating speckle patterns and insufficient camera frame rate.

**Figure 9:**
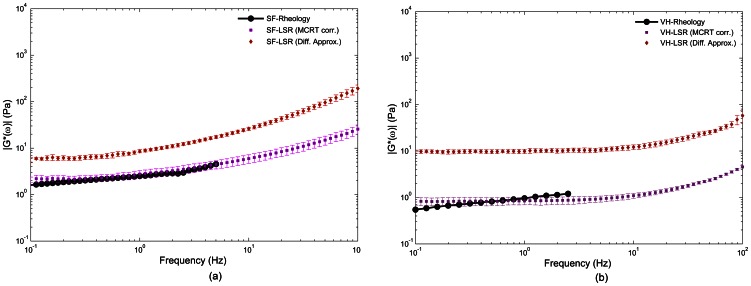
LSR results of |G*(ω)| for synovial fluid and vitreous humor measured with and without optical scattering correction. The red diamonds are the average |G*(ω)| moduli, of synovial fluid (panel (a)) and vitreous humor (panel (b)) samples of Fig. 8, obtained from LSR by using the DWS expression (eqn. (3)). The red error bars correspond to standard error values. The purple squares represent the average |G*(ω)| moduli, obtained from the corrected MSD values using eqn. (6), and the purple error bars correspond to the standard errors. Also depicted in this figure are the |G*(ω)| values for the samples measured using a conventional rheometer (black solid line, round markers). While LSR results compensated for optical scattering show close correspondence with rheology measurements, the DWS-based approach results in an offset of about one decade relative to conventional testing results.

Figs. 8 (a) and (b) show *g_2_^exp^(t)* curves measured from time-varying speckle images of synovial fluid and vitreous humor, respectively. Similar to the glycerol samples, the *g_2_^exp^(t)* decay accelerated with increased scattering in both cases. Since *g_2_^exp^(t)* decayed slower for synovial fluid compared to vitreous humor, it was expected that synovial fluid would have a relatively higher modulus. However, it was necessary to correct for the contribution of optical scattering prior to comparing absolute mechanical moduli. Fig. 9 shows the LSR results of |G*(ω)| for synovial fluid (Fig. 9a) and vitreous humor (Fig. 9b) measured with and without optical scattering correction. The red diamonds represent average |G*(ω)| values of synovial fluid and vitreous humor samples of Fig. 8, estimated using LSR based on the DWS expression (eqn. (3)) which did not take into account optical scattering variations. The purple squares correspond to the moduli resulted from corrected MSD values, using the modified expression of eqn. (6), derived from the compensation algorithm. The red and purple error bars stand for the standard error. Also depicted in this figure are the |G*(ω)| values measured using a conventional rheometer (black solid line, round markers). It was evident that in the case of LSR with optical scattering correction, |G*(ω)| exhibited a close correspondence with conventional mechanical testing. Moreover, |G*(ω)| measured using DWS approximation resulted in an offset of about one decade relative to conventional rheometric testing results. This was due to slower decay of speckle intensity temporal autocorrelation curve, caused by relatively low concentration of TiO_2_ particles as discussed later. From the results of Fig. 9, it was clear that synovial fluid had a slightly higher viscoelastic modulus, which was consistent with our initial observation of speckle fluctuations and with standard reference mechanical rheometry. Moreover, the non-Newtonian behavior of these bio-fluids, reflected in smaller slope of |G*(ω)| and lower frequency dependence compared to viscous glycerol solutions, pointed to the complex viscoelastic behavior of bio-fluids relative to glycerol samples. These results established the critical need of compensating for optical scattering properties to enable accurate measurement of viscoelastic moduli from laser speckle patterns and demonstrated the potential of LSR for evaluating the viscoelastic properties of biological fluids.

## Discussion

In this study, we have developed a new approach to significantly improve the accuracy of LSR for evaluating the viscoelastic properties of bio-fluids by correcting for the influence of optical scattering on laser speckle intensity fluctuations. We anticipate that this work will potentially open new avenues for the application of LSR in clinical diagnosis, treatment monitoring, tissue engineering, and drug development.

In LSR it is critical to correctly deduce the MSD of scattering particles from speckle fluctuations, given by *g_2_(t)*, to derive |G*(ω)| (eqn. (7)) [18]–[20], [22], [23], [25], [26], [36]–[41]. The major difficulty, however, is that *g_2_(t)* curve not only depends on the sample viscoelasticity but also on its optical properties, which define light transport in the medium. Thus, LSR evaluation of viscoelastic properties is particularly challenging in bio-fluids which span a range of optical properties. For instance, aqueous humor and vitreous humor are almost transparent, cerebrospinal fluid, synovial fluid, plasma, and lymph are moderately scattering, and mucus, sputum, milk, blood and bile are highly scattering and absorbing at different wavelengths. Furthermore, the optical (and mechanical) properties of bio-fluids are often altered in diseased states, which are of particular interest.

Previously, DLS and DWS approximations have been used to derive MSD from *g_2_(t)*. In DLS, the well-defined scattering geometry and single scattering assumption simplify the *g_2_(t)* to an exponentially decaying function of MSD [32]. In the other extreme, using DWS, light diffusion theory approximates the path length distribution and provides an expression for *g_2_(t)* [32]. However, in moderately scattering samples and for small source-detector distances, DLS and DWS cannot be directly applied to the analysis of MSD (Figs. 5(a) & (b)). The *g_2_(t)* curves of glycerol samples, displayed in Fig. 4 demonstrate that for samples of identical mechanical properties, *g_2_(t)* can still be modulated by tuning optical scattering properties and in a moderately scattering material lower numbers of scattering counts can result in a *g_2_(t)* curve that decays slower compared to a rich scattering medium of similar viscosity (Fig. 4). We have shown that the direct use of DWS formalism leads to an underestimation of the MSD (Fig. 5a), and in turn results in inaccurate |G*(ω)| values biased by the optical scattering concentration (Fig. 5(b)). Similarly, if DLS approximation is used, it may overestimate the MSD because for moderately scattering media, speckle intensity temporal autocorrelation curve decays faster compared to a single scattering scenario [48]. Therefore, a more precise *g_2_(t)* - MSD expression is required to account for variations in optical properties in order to estimate accurate |G*(ω)| values using LSR.

Leveraging our earlier work, absorption and reduced scattering coefficients (*μ_a_*, *μ′_s_ = μ_s_*×(1-*g*)) were similarly evaluated in this study from the photon flux profile (Fig. 6(a)), obtained from temporally averaged speckle image series [14]. The reduced scattering coefficient, *μ′_s_*, measured using this method demonstrated close agreement with theoretical calculations particularly at low and intermediate scattering concentrations. Deviations at higher concentrations were likely caused by clumping (larger particles, larger g) and sedimentation of TiO_2_ particles (given the density of 4.23 g/cm^3^ for TiO_2_ relative to <1.26 g/cm^3^ for glycerol mixture) which resulted in lower *μ′_s_* compared to Mie predictions. Moreover, at higher TiO_2_ concentrations close proximity of adjacent particles could also lead to interactions of near-field radiation and reduce the backscattering efficiency, which influenced the measured *μ′_s_* values [32], [64], [65]. For predominantly scattering samples, used here, results were solely focused on the influence of *μ′_s_* variations on the speckle dynamics and the role of absorption was not studied. Nonetheless, optical absorption is expected to eliminate rays with longest optical paths, corresponding to a large number of scattering events, and decelerate *g_2_(t)* curves [32]. In the received back-scattered signal, attributes of scattering angular distribution were extensively washed off by multiple scattering. As a result, experimental evaluation of phase function and *g* was not trivial and instead theoretical Mie calculations were used to predict these parameters which resulted in *g* = 0.6 for TiO_2_ particles suspended in glycerol suspensions. Thus, in the current study, the effect of scattering anisotropy was not addressed in experiments. In the future application of LSR in bio-fluids, in their native states, the typical values of scattering asymmetry parameter for tissues (*g*  = 0.7–0.9) can be used [53]. Theoretical analyses by others indicate that for fixed *μ′_s_* and particle size values, the changes in *g* have minimal effect on the *g_2_(t)* trend [43], [61]. Our PSCT-MCRT simulations of *g_2_^MCRT^(t)* could independently confirm these findings. In reality, however, the influence of the anisotropy parameter cannot be isolated from the MSD. This is because *g* is directly related with scattering particle size, *a*, and in turn with the MSD. For instance, larger particles have a larger *g* value, and also exhibit slower Brownian displacements, i.e. small MSD, and slow down the *g_2_(t)* trend.

As shown in Fig. 6(b), experimentally measured *μ′_s_* values were validated via comparison with corresponding values obtained from Mie theory predictions that assumed near identical size spheres [56]. A DLS-based particle sizer (Zeta Sizer, Malvern Instruments, USA) was used to determine particle size, which provided an average hydrodynamic diameter of 400 nm and a polydispersity index (PdI, i.e. normalized distribution width) of 0.3 for the TiO_2_ particles used in our experiments. Given the low PDI of 0.3 (below 0.5), the particles could be considered sufficiently mono-dispersed for Mie theory approximation. The TiO_2_ particles were non-spherical pyramidal crystals [66]. However, due to their small size-parameter, (radius times wave number, *kr*<<5) and random orientations in the suspension, the use of Mie theory predictions could be further justified [67], [68].

The PSCT-MCRT model used here incorporated the experimental configurations of the LSR setup (Fig. 1), the finite sample geometry, and measured optical properties to derive a modified expression that related *g_2_(t)* with MSD in samples that do not meet the criteria of single scattering or diffusive regime. To this end, at first speckle field temporal autocorrelation function, *g_1_(t)* was derived in terms of MSD, as shown by the term in brackets in eqn. (4). Next, the Siegert relation [27], [28], [35], 44,62 was used to express *g_2_(t)* in terms of *g_1_(t)* and MSD, as shown in eqns. (4) and (6) and the flowchart of Fig. 2. The modified expression of eqn. (6) converged to the classical DLS and DWS expressions (eqns. (2 and 3)) in the limits of single and rich multiple scattering, respectively, as explained in the materials and methods section. The parameter, *γ*, in the above equations is an scattering dependent variable, governed by the sample optical properties, concentration of light scattering particles, the polarization state of collected back-scattered light, reflectivity of samples walls (i.e. boundary conditions), and other experimental parameters [69]–[71]. In the limit of strongly scattering medium, the *γ* is traditionally assumed to be 5/3 [17], [19], [21]–[23], [28], [44], [45]. Consistent with this assumption, our MCRT results exhibited the asymptotic value of *γ* = 5/3 in the limit of rich, multiple scattering samples. The MSD values (Fig. 7(a)), estimated using the PSCT-MCRT-derived expression, demonstrated significant improvement compared with the DWS-based approach in compensating for scattering variations. The minimal residual MSD variability at early times was likely due to CMOS sensor noise, pronounced in the few initial points of *g_2_(t)* curve particularly in low scattering samples with lower speckle intensity. Deviations at long times of Fig. 7(a) were caused by rapid speckle dynamics and insufficient camera frame rate which led to spatio-temporal speckle blurring and an artificial plateau in *g_2_(t)* curve which was transferred to long time MSD slope. This minor hardware limitation could be avoided by exploiting higher frame rates and maintaining sufficient signal to noise ratio. The |G*(ω)| curves of Fig. 7(b), estimated from the corrected MSD values, showed high correspondence with the results of mechanical rheometry. The variability between measured |G*(ω)| for different concentrations was considerably reduced and the absolute value of the |G*(*ω)*| curves converged to that obtained using reference-standard mechanical testing. The minimal scattering-dependent discrepancy in the |G*(ω)|, at low and high frequencies, resulted from initial and long times variations of MSD as explained above.

Similarly, in the low viscosity bio-fluids, studied here, LSR measurements of |G*(ω)| at low frequencies (∼0.1 Hz) were influenced by speckle blurring caused by insufficient camera frame rate. These effects were minimized by subtracting the background signal (caused by speckle blurring) to improve speckle contrast prior to evaluation of *g_2_(t)* (Fig. 8). With these corrections, LSR could measure viscoelastic behavior over a frequency range that spanned three decades (0.1–100 Hz). High frame rate speckle acquisition could potentially extend this frequency range through probing speckle dynamics and sample mechanics at finer temporal resolution and consequently higher frequencies. Also, it would improve speckle contrast and extend the validity of LSR results to lower oscillation frequencies and enable probing structural and rheological behavior of bio-fluids in more details. For these bio-fluids, constraints of rotational rheometer for evaluating the |G*(ω)| at high oscillation frequencies limited the comparison of LSR with mechanical rheometry to below 10 Hz (Figs. 9(a) and (b)). At higher frequencies, the torque (stress) applied by the rheometer motor shaft to rotate the rod and top plate rapidly increased with frequency (due to inertia) and exceeded the torque needed to strain the low viscosity bio-fluids. This is an inherent limitation of mechanical rheometer that cause unreliable readouts of |G*(ω)| at higher frequencies.

In the current LSR setup, focused beam illumination and full-field collection led to a broad distribution of optical path lengths for received rays, with each length probing a different time-scale (frequency) of the sample dynamics [32]. The multi-speckle collection enabled shorter acquisition and better statistical accuracy by exploiting both ensemble and temporal averaging (eqn.(1)) [49], [72]. To permit depth-resolved mapping of bio-fluids' mechanical properties, spatio-temporal processing of speckle patterns can be employed as previously described [14]. Low coherence interferometery (e.g. M-mode OCT) techniques can also probe particle dynamics in specific depths within the medium with superior resolution and potentially evaluate the viscosity [29], [30]. However, there exists a tradeoff between higher depth-resolution capabilities of OCT versus higher measurement sensitivity of LSR to particles' displacements (MSD). This is because in LSR the detected light has scattered multiple times over a volume of interest. Therefore, even minute motions (fraction of a wavelength) of particles give rise to cumulative phase changes over an ensemble of light paths within the measured volume and induce detectable reduction in speckle intensity temporal autocorrelation. By measuring *g_2_(t)* over multiple speckles as in LSR, particle displacements as small as a few angstroms can be detected [32], [37], [73]. In OCT however, single scattered light is detected. Therefore, a substantially larger displacement of particles is required to induce a sufficient path length change and noticeable reduction of speckle intensity temporal autocorrelation particularly in highly viscous media with smaller particle MSD [32], [37], [72], [73].

As described earlier, in the current work TiO_2_ particles were used to sufficiently validate our approach by tuning the reduced scattering coefficient over a range of scattering concentrations. Adding the extrinsic scattering particles, at various arbitrary concentrations enabled complete evaluation of scattering variations effects, and validation of the proposed compensation algorithm. In future, we anticipate that LSR will be used to measure bio-fluid viscoelasticity in the native state without addition of extrinsic light scattering particles. This will require additional information about particle size parameter, *a*, to be evaluated experimentally. To this end, the future LSR configuration may be coupled with polarization dependent analysis of diffused back-scattered light or angle-resolved detection of low coherence radiation to enable particle sizing and permit the quantification of G*(ω) of bio-fluid in their native states [74]–[77]. In addition, LSR can potentially be conducted via needle-based probes or endoscopes to enable future *in vivo* use. By limiting the illumination and collection volume at the interrogation site residual scattering from surrounding structures could be potentially restricted. We anticipate that the demonstrated capability of LSR for the non-contact and accurate evaluation of viscoelastic properties and the potential of this technology in probing rheological properties of bio-fluids will open multiple new avenues for clinical applications of LSR in the future.
